# Supplementary material for: Spatial transcriptomics inferred from pathology whole-slide images links tumor heterogeneity to survival in breast and lung cancer
Source: Sci Rep. 2020 Nov 2;10:18802. doi: 10.1038/s41598-020-75708-z (PMC7606448; doi:10.1038/s41598-020-75708-z)
Supplement: Supplementary file 1 — Supplementary Table 1. [file 41598_2020_75708_MOESM1_ESM.pdf]

Spatial Transcriptomics Inferred from Pathology Whole-Slide Images Links Tumor Heterogeneity to Survival in Breast and Lung Cancer

Alona Levy-Jurgenson[1,*], Xavier Tekpli[2,3], Vessela N. Kristensen[2,3,4], and Zohar Yakhini[1,5*]

[1]Department of Computer Science, Technion - Israel Institute of Technology, Haifa 32000, Israel
[2]Department of Medical Genetics, Institute of Clinical Medicine, University of Oslo and Oslo University Hospital, Oslo, Norway
[3]Department of Cancer Genetics, Institute for Cancer Research, Oslo University Hospital, 0310 Oslo, Norway
[4]Department of Clinical Molecular Biology and Laboratory Science (EpiGen), Division of Medicine, Akershus University Hospital, Lørenskog, Norway
[5]Arazi School of Computer Science, Interdisciplinary Center, Herzliya 4610101, Israel
[*]Correspondence to: levy.alona@gmail.com and zohar.yakhini@gmail.com

Supplementary Material

Supplementary Note S1

Table S1. Spearman correlations and FDR-corrected p-values for breast and lung cohorts across test, OOD-near and OOD-all data sets. Slides are scored using the percent positively classified tiles produced by the ensemble model for each trait. Ground-truth values are the actual slide percentiles (0.1, 0.2 etc.). In bold are significant FDR-corrected p-values ≤ 0.1.

	**Gene / Trait**	**Test**		**OOD-near**		**OOD-all**	
		**Spearman**	**p-value (FDR)**	**Spearman**	**p-value (FDR)**	**Spearman**	**p-value (FDR)**
TCGA-BRCA	miR-17-5p	**0.63**	**8e-04**	**0.21**	**0.02**	**0.13**	**0.01**
	MKI67	**0.58**	**1e-03**	**0.38**	**4e-06**	**0.27**	**6e-08**
	FOXA1	**0.44**	**0.04**	**0.19**	**0.03**	**0.12**	**0.01**
	MYC	0.36	0.11	**0.16**	**0.07**	**0.13**	**0.01**
	miR-29a-3p	0.38	0.11	**0.27**	**4e-03**	**0.13**	**0.01**
	ESR1	0	1.00	**0.43**	**5e-07**	**0.21**	**3e-05**
	CD24	0.10	0.93	0.12	0.17	8e-03	0.86
	FOXC1	4e-03	1.00	**0.31**	**3e-04**	**0.21**	**3e-05**
	ERBB2	0.07	0.99	0.03	0.70	0.02	0.72
	EGFR	0.05	1.00	0.12	0.16	**0.12**	**0.02**
TCGA-LUAD	miR-17-5p	0.56	0.22	-1e-01	0.40	1e-03	0.98
	KRAS	0.38	0.32	0.11	0.40	0.03	0.74
	CD274 (PD-L1)	0.39	0.39	**0.23**	**0.10**	0.07	0.43
	miR-21-5p	0.17	0.49	**0.43**	**2e-04**	**0.24**	**4e-04**
	EGFR	0.27	0.49	-3e-02	0.78	-7e-02	0.43
